# Robust Mesh Segmentation Using Feature-Aware Region Fusion †

**DOI:** 10.3390/s23010416

**Published:** 2022-12-30

**Authors:** Lulu Wu, Yu Hou, Junli Xu, Yong Zhao

**Affiliations:** 1 School of Mathematical Sciences, Ocean University of China, Qingdao 266100, China; 2 School of Mathematics and Physics, Qingdao University of Science and Technology, Qingdao 266061, China

**Keywords:** 3D meshes, feature-aware region fusion, robust segmentation

## Abstract

This paper introduces a simple but powerful segmentation algorithm for 3D meshes. Our algorithm consists of two stages: over-segmentation and region fusion. In the first stage, adaptive space partition is applied to perform over-segmentation, which is very efficient. In the second stage, we define a new intra-region difference, inter-region difference, and fusion condition with the help of various shape features and propose an iterative region fusion method. As the region fusion process is feature aware, our algorithm can deal with complex 3D meshes robustly. Massive qualitative and quantitative experiments also validate the advantages of the proposed algorithm.

## 1. Introduction

In computer vision, computer graphics, and multimedia processing, the segmentation of 3D meshes is a popular but difficult research topic. As an important operation, mesh segmentation is helpful for conducting 3D analysis and developing our understanding, and can be applied to various subsequent operations. For example, segmentation results can provide semantic information for high-level feature extraction. Moreover, the user can first segment a variety of 3D meshes. Then, some mesh parts from different meshes can be composed to generate new 3D meshes. Therefore, the accuracy and robustness of segmentation algorithms are indispensable.

The purpose of mesh segmentation is to decompose an input 3D mesh into multiple semantic parts. The key is how to make full use of its shape features to obtain a perception-aware segmentation result. Three-dimensional meshes often have complex shapes or rich details, which makes mesh segmentation very challenging. Many works focus on automatic segmentation algorithms [1,2,3]. Interactive segmentation algorithms [4,5] guide the segmentation process through user-specified strokes. In recent years, data-driven methods have been used to deal with the mesh segmentation problem [6,7,8]. In particular, deep learning techniques have been employed to enhance the generalization ability [9,10,11,12]. However, segmentation accuracy still needs to be improved for practical applications.

**Figure 1:**
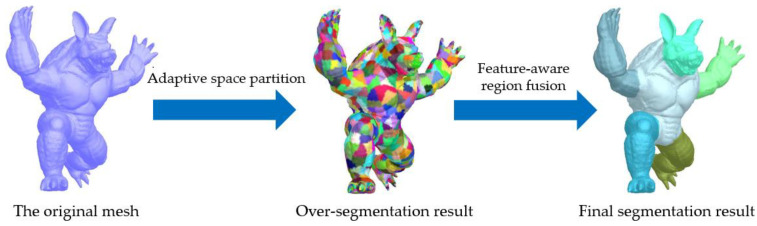
Algorithm overview conducted on an armadillo mesh [6]. Firstly, we perform a rapid over-segmentation using the adaptive space partition to yield a set of superfacets. Secondly, we use a feature-aware region fusion method to merge similar superfacets to generate the final segmentation result. For demonstration, all superfacets and segmentation parts are rendered with random colors.

To address the mesh segmentation problem robustly, we developed a feature-aware algorithm that utilizes various shape features of 3D meshes and can obtain perception-aware results efficiently. Figure 1 shows the overview of the algorithm conducted on a mesh with a complex shape and rich details. The input mesh is large and the segmentation run-time depends nonlinearly on mesh size, as an input mesh may have millions of facets. Therefore, we group input facets into collections of contiguous mesh facets; each collection is called a superfacet. There are about 40× fewer superfacets than input facets; thus, we perform segmentation on these superfacets for a speed up to about 100×.

Then, a variety of shape features are calculated to represent local attributes of superfacets, such as normal, Gaussian curvature [13], shape diameter function [14], average geodesic distance [15], conformal factor [16], and heat kernel signature [17] attributes. We further introduce an intra-region difference, inter-region difference, and fusion condition, and iteratively fuse neighboring superfacets with similar attributes to generate a segmentation result.

In order to validate the effectiveness and robustness of our algorithm, segmentation experiments are conducted on a large number of 3D meshes with complex shapes or rich details. Even if Gaussian noises, holes, missing parts, pose changes, or sampling changes appear on the 3D meshes, our algorithm can still obtain visually pleasing results. Therefore, we can qualitatively and quantitatively compare it with many state-of-the-art methods. Quantitative metrics for the evaluation of the segmentation results include accuracy, Rand Index, Cut Discrepancy, Consistency Error, and Hamming Distance. All the comparisons demonstrate the advantages of our algorithm. The proposed algorithm has several parameters. We tune them manually to obtain the best result.

The main contributions of this paper can be summarized by the following two points:

1. An efficient over-segmentation method is introduced via adaptive space partition.
2. By defining a new intra-region difference, inter-region difference, and fusion condition, a simple but powerful feature-aware region fusion algorithm is proposed that can robustly achieve mesh segmentation.

## 2. Related Work

Many automatic segmentation algorithms have been proposed. Chazelle et al. [18] presented a method for decomposing a polygonal surface using a flooding heuristic. Zhou and Huang [19] decomposed a polygon mesh into meaningful parts or regions by means of critical points. Katz et al. [1] addressed mesh segmentation through fuzzy clustering and graph cuts. Lavoue and Wolf [20] presented a mesh segmentation method based on Markov random fields. Golovinskiy et al. [21] adopted randomized cuts for mesh segmentation and analysis. A 3D mesh segmentation benchmark, proposed by Chen et al. [22], can analyze and compare different segmentation algorithms quantitatively. Au et al. [2] introduced a mesh segmentation algorithm using concavity-sensitive scalar fields. Theologou et al. [23] constructed a heterogeneous graph containing local features and patch affinities and performed spectral partitioning with the help of nodal set and nodal domain theory. Zhang et al. [24] obtained their initial result via spectral clustering, and then discretized the Mumford–Shah model for further refining. Tong et al. [3] formulated mesh segmentation as a L_0_ optimization problem with respect to the Fiedler vector. Zhang et al. [25] segmented a mesh by blending regions into different patches and fitted each patch through a surface primitive. Lin et al. [26] performed segmentation based on a medial axis transform, which encodes both geometrical and structural information.

Interactive segmentation algorithms are employed to reflect user intentions. Ji et al. [4] drew a foreground stroke and a background stroke on the mesh model for guidance. Then, the segmentation result can be generated via region growth. Later on, various interaction tools were presented. Zheng et al. [27] introduced cross-boundary strokes to identify the desired cut. Fan et al. [28] only specified a stroke on the foreground region. Zheng et al. [5] only needed to draw a boundary point. Further, Hou et al. [29] combined random walks with the L_0_ constraint to locate segmentation boundaries.

Recently, data-driven methods have been adopted to deal with the segmentation problem. Based on some labeled meshes, Kalogerakis et al. [6] applied a conditional random field model to learn the segmentation results. Benhabiles et al. [7] used the AdaBoost classifier to learn the boundaries between mesh parts. Wang et al. [8] projected a 3D model onto a set of binary images, found the initial labels in a labeled image set, and optimized them by using the graph cut method.

Subsequently, deep neural networks can be utilized to improve the generalization ability. Guo et al. [9] organized mesh features into 2D matrices and used these data as the input of the network to learn the facets’ labels. George et al. [12] and Shu et al. [30] used feature vectors as the network input to reduce the mutual influence among different features. Taking multi-view rendered images and depth images as the input, Kalogerakis et al. [10] trained convolutional neural networks and a conditional random field in an end-to-end way. To handle the unstructured sampling of mesh models, Xu et al. [11] defined rotation-invariant convolution and pooling operations. Yi et al. [31] generated segmentation results by combining the neural network and spectral analysis. Wang et al. [32] investigated the segmentation problem by using volumetric representation and proposed a voxel-based feature extraction module and an attention-based feature aggregation module. Hu et al. [33] operated on both voxels and a mesh surface to incorporate Euclidean information and geodesic information. Additionally, weakly supervised and semi-supervised schemes were employed to reduce the requirements of the training data [34,35].

Some segmentation methods were designed for medical treatment. Xu et al. [36] aimed to cut out each tooth in a 3D dental model. Lawonn et al. [37] performed segmentation on the 3D surfaces of vessels and then identified aneurysms. As an extension, the segmentation of point clouds has drawn a lot of attention. For example, Wang et al. [38] introduced a similarity group proposal network for instance segmentation on point clouds. Furthermore, the segmentation problem is closely related to many other 3D processing problems, such as object classification [39], shape unfolding [40], and surface denoising [41,42].

Our main idea was published during the CDMMS2020 [43]. This paper presents substantial improvements over the previous short conference version in both the algorithm and experiments. Generally, we improve our algorithm (e.g., the intra-region difference, the inter-region difference, and the threshold function), and provide further analysis and discussion, which explains why the proposed algorithm works well. Firstly, the intra-region difference is defined as the maximum of the differences between adjacent superfacets in [43]. As the maximum would overestimate the difference, we adopt the average in this paper. Secondly, the inter-region difference is defined as the minimum of the differences at the boundary in [43]. Considering that the minimum would underestimate the difference, we also adopt the average in this paper. By contrast, our new definitions are more reasonable and more effective for handling different kinds of 3D meshes. Thirdly, the threshold function is based on the formulation of $\frac{1}{x}\;$ in [43]. We adopt the formulation of $\frac{1}{e^{x}}\;$ in this paper. As proved by the existing algorithms of [29,44,45], the formulation of $\frac{1}{e^{x}}$ is more flexible when dealing with complex cases. Additionally, in our prior paper [43], we only conducted limited segmentation experiments. In this paper, we provide comprehensive segmentation results, and further evaluate the robustness in regard to Gaussian noises, holes, missing parts, pose changes, and sampling changes. We also compare our algorithm with existing state-of-the-art algorithms qualitatively and quantitatively to show our advantages, and then demonstrate its high-time performance.

## 3. Feature-Aware Mesh Segmentation Algorithm

A 3D mesh consists of mesh vertices and triangular facets. Each mesh vertex has a 3D position. Three vertices form a triangular facet. In this section, we will elaborate the proposed segmentation algorithm, including the binary space partition and feature-aware region fusion.

### 3.1. Efficient Adaptive Space Partition

Three-dimensional meshes usually contain a large number of triangular facets, and exhibit complex shapes or rich details. If the segmentation is performed on triangular facets, it would lead to a low-time performance. Hence, binary space partition [46] is adopted to split a 3D mesh into a set of superfacets, which could reduce computational costs and improve time performance. Suppose $p_{i}\left(1\leq i\leq k\right)$ is a mesh vertex, and k is the number of vertices in the current partition space; then, the 3 × 3 covariance matrix can be expressed as:

$$C={\left[\begin{array}{c} p_{1}-\bar{p}\\ \;\cdots \;\\ p_{k}-\bar{p} \end{array}\right]}^{T}\cdot \left[\begin{array}{c} p_{1}-\bar{p}\\ \;\cdots \;\\ p_{k}-\bar{p} \end{array}\right],$$

where $\bar{p}\;$ is the centroid of all vertices in the current partition space.

The eigen equation of  C can  be expressed as: $C\cdot v_{l}=\lambda_{l}\cdot v_{l}$, where l∈{0,1,2}, $\lambda_{l}$ is an eigenvalue, and $v_{l}$ is an eigenvector. $\lambda_{l}$ is a good measure of the variation in the mesh along the direction of $v_{l}$. Assuming $\lambda_{0}\leq \lambda_{1}\leq \lambda_{2}$, according to [46], the surface variation is defined as follows:

$$\sigma =\frac{\lambda_{0}}{\lambda_{0}+\lambda_{1}+\lambda_{2}}$$

Closely related to the mean curvature, this surface variation can effectively estimate the characteristics of the mesh.

The partition plane is defined as follows:

$$P\left(x\right):\;\left(x-\bar{p}\right)\cdot v_{0}=0,$$

where $v_{0}$ is the corresponding eigenvector of $\lambda_{0}$. In other words, we always partition the mesh model along the direction of the greatest variation.

In practice, we perform binary space partition recursively. Each partitioned subspace should be further partitioned until its number of triangular facets is less than the threshold $q_{max}$ and its surface variation is less than the threshold $\sigma_{max}$. When this process is finished, the triangular facets in each partitioned subspace are treated as a superfacet. Please note that the number of superfacets is far less than that of triangular facets. We denote the set of superfacets as $F=\left\{F_{i},1\leq i\leq \left|F\right|\right\}$, where $F_{i}$ is a superfacet, and |F| is the number of superfacets.

**Figure 2:**
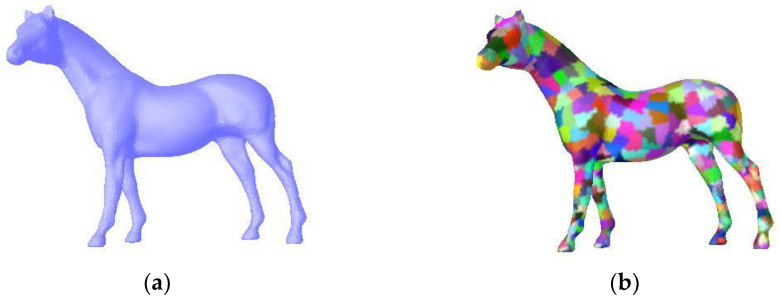
Over-segmentation carried out on a horse mesh. (**a**) The original mesh. (**b**) Over-segmentation result.

Figure 2 shows an example of over-segmentation. The horse model has 48,485 vertices and 96,966 facets. Due to the high efficiency of binary space partition, it only takes 0.094 s to complete the over-segmentation. This example produces 1218 superfacets. Thus, performing segmentation on superfacets greatly reduces computational cost and improves time efficiency.

Previous methods pay little attention to over-segmentation, and fulfill this task via existing segmentation algorithms. Algorithm parameters are tuned to generate superfacets rather than mesh parts. However, over-segmentation is very important for mesh segmentation. We introduce a very efficient over-segmentation scheme through adaptive space partition.

### 3.2. New Feature-Aware Region Fusion

#### 3.2.1. Feature Description

We can describe local mesh attributes via various shape features. For example, normal, Gaussian curvature [13], shape diameter function [14], average geodesic distance [15], conformal factor [16], and heat kernel signature [17]. These shape features are computed for each superfacet $F_{i}$. As the above features have different scales, we first normalize each shape feature separately, and then concatenate them to form a feature vector $T\left(F_{i}\right)$ for superfacet $F_{i}$. We further define the difference between adjacent superfacets $F_{i}$ and $F_{j}$ as follows:

(1) $$d\left(F_{i},F_{j}\right)=∥T\left(F_{i}\right)-T{\left(F_{j}\right)∥}_{2}$$

where ${∥\cdot ∥}_{2}$ is the L_2_ norm. In other words, the difference is the Euclidean distance between feature vectors.

#### 3.2.2. Region Fusion

Initially, each superfacet represents a local region. Region fusion is an intuitive method that fuses these regions together to obtain the final segmentation result. Two adjacent regions have similar attributes and should belong to the same part of the model if their difference is relatively small. Under this condition, we fuse them into one region. In the following, we elaborate our intra-region difference, inter-region difference, fusion condition, and fusion process.

Suppose R is a local region that consists of several superfacets. Its intra-region difference is defined as the average of the differences between adjacent superfacets. That is,

(2) $$D\left(R\right)=\underset{F_{i},F_{j}\in R}{\mathrm{Average}}\left\{d\left(F_{i},F_{j}\right)\right\},$$

where $F_{i}$ and $F_{j}$ are two adjacent superfacets in R. Our prior paper [43] used the maximum, which would overestimate the difference. Obviously, this new definition can reflect surface changes in R more effectively.

Suppose $R_{1}$ and $R_{2}$ are two adjacent regions. We define their inter-region difference as the average of the differences at the boundary:

(3) $$Dis\left(R_{1},R_{2}\right)=\underset{F_{i}\in R_{1},F_{j}\in R_{2}}{\mathrm{Average}}\left\{d\left(F_{i},F_{j}\right)\right\},$$

where $F_{i}$ and $F_{j}$ are two adjacent superfacets from $R_{1}$ and $R_{2}$, respectively. Our prior paper [43] used the minimum, which would underestimate the difference. In contrast, this new definition is more reasonable for measuring geometric changes between $R_{1}$ and $\;R_{2}$.

Two adjacent regions can be fused into a new region if their inter-region difference is less than the minimum value of their intra-region differences. We formulate this fusion condition as:

(4) $$Dis\left(R_{1},R_{2}\right)\leq \mathrm{Min}\left\{D\left(R_{1}\right)+t\left(R_{1}\right),D\left(R_{2}\right)+t\left(R_{2}\right)\right\},$$

where $D\left(R_{1}\right)$ and $D\left(R_{2}\right)$ are the intra-region differences of $R_{1}$ and $R_{2}$, respectively, and t(⋅) is a threshold function with respect to local regions. t(⋅) is used to adjust the above fusion condition. Initially, each local region contains only one superfacet; thus, $D\left(R_{1}\right)=0$, and $D\left(R_{2}\right)=0$. The fusion process would fail to start if there is no t(⋅) in Equation (4).

When there are fewer superfacets in the region, t(⋅) should have a relatively large value to relax the fusion condition. When the regions contain more superfacets due to fusing, t(⋅) should be closer to 0 and should have little effect on the fusion process. Therefore, t(⋅) should be a decreasing function, which decreases as the number of superfacets in the region increases. As proved by the existing algorithms of [29,44,45], the formulation of $\frac{1}{e^{x}}$ works well in practice. In this paper, our threshold function is defined as:

(5) $$t\left(R\right)=\frac{m}{1+e^{\left|R\right|}},$$

where |R| is the number of superfacets in the local region R, and m is a constant. The prior threshold function is based on the formulation of $\frac{1}{x}\;$ in [43]. In comparison, the new threshold function is more flexible for handling complex cases.

All inter-region differences of the adjacent regions are sorted from small to large. Then, the fusion is performed according to this order. After the two regions are fused, we update their intra-region differences and inter-region differences with other adjacent regions. This fusion process will only stop if all adjacent regions fail to meet the fusion condition

#### 3.2.3. Boundary Smoothing

When the above fusion is finished, if a region contains few superfacets, we will merge it into an adjacent region with the smallest inter-region difference. Furthermore, there might be jaggy boundaries between adjacent regions. The graph cut algorithm [47] is applied to smooth these boundaries. Without a loss of generality, we discuss this process between two adjacent regions, $R_{1}$ and $R_{2}$. We grow a narrow fuzzy region S from their current boundary. Those facets that directly connect to $R_{1}$ or $R_{2}$ are collected as set M or set N, respectively. For any facet $f_{i}$ in S, its unit normal and local neighborhood are denoted as $n_{f_{i}}$ and $N_{i}$, respectively. Additionally, we use $R_{f_{i}}$ to represent which region $f_{i}$ belongs to. In other words, $R_{f_{i}}$ should be either $R_{1}$ or $R_{2}$.

**Figure 3:**
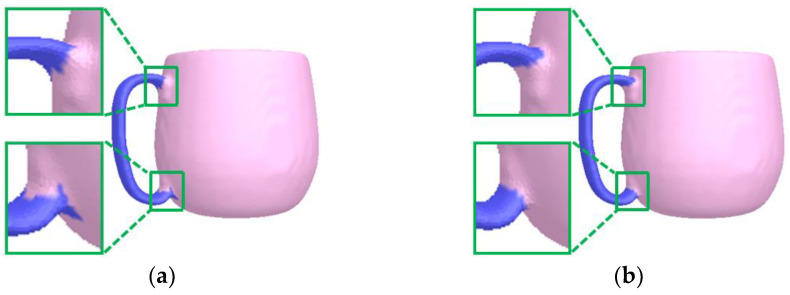
Segmentation boundary smoothing. Please note that jaggy boundaries become smoother. (**a**) Boundaries before smoothing. (**b**) Boundaries after smoothing.

The graph cut algorithm needs to optimize an energy function consisting of a data term and a regularization term. The data term is defined as:

$$\sum_{f_{i}\in S}^{\;}E_{d}\left(R_{f_{i}}\right),$$

where $E_{d}\left(R_{f_{i}}\right)=\{\begin{array}{c} 1,\;if\;R_{f_{i}\;}conflicts\;with\;M\;and\;N\\ 0,\;otherwise \end{array}$. The regularization term is defined as:

$$\sum_{f_{i}\in S}^{\;}\sum_{f_{j}\in N_{i}}^{\;}w_{ij}E_{r}\left(R_{f_{i}},R_{f_{j}}\right),$$

where $E_{r}\left(R_{f_{i}},R_{f_{j}}\right)=\{\begin{array}{c} 1,\;R_{f_{i}}\neq R_{f_{j}}\\ 0,\;R_{f_{i}}=R_{f_{j}} \end{array}$, $w_{ij}=e^{-{∥n_{f_{i}}-n_{f_{j}}∥}_{2}}$, $n_{f_{j}}$ is the unit normal of $f_{j}$, and $R_{f_{j}}$ represents which region $f_{j}$ belongs to. $w_{ij}$ describes the similarity between $f_{i}$ and $f_{j}$. The larger it is, the more likely it is that $f_{i}$ and $f_{j}$ should belong to the same region. As shown in the close-up views in Figure 3, this scheme is able to smooth region boundaries.

**Figure 4:**
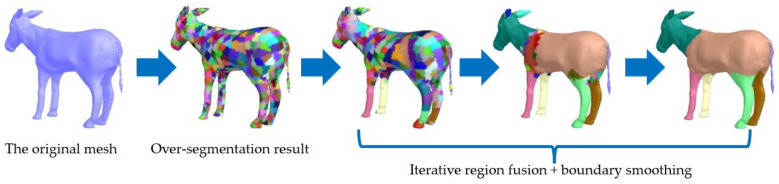
Feature-aware segmentation process.

Finally, each region forms a segmentation part of the mesh. Figure 4 shows the segmentation process of our algorithm. First, adaptive space partition is used to obtain an over-segmentation result. Then, regions that meet the fusion condition are iteratively merged to obtain the final segmentation result.

## 4. Experiments and Discussions

### 4.1. Results and Analysis

We implemented our algorithm by using C++ on a computer with an Intel Core i5-5200U CPU. There are three parameters of this algorithm: $\left\{q_{max},\;\sigma_{max},\;m\right\}$. The parameters $q_{max}$ and $\sigma_{max}$ are used to control over-segmentation. Here, $q_{max}\in$ [10, 80]. The default value for $\sigma_{max}$ is 0.1. The parameter m is used to adjust t(⋅) to control the fusion condition, and m∈[2, 12]. The segmentation experiments were performed on a large number of 3D meshes, some of which have Gaussian noises, holes, missing parts, pose changes, or sampling changes. In addition, we also made qualitative and quantitative comparisons with some previous state-of-the-art methods. All these results validate the advantages of the proposed algorithm.

**Figure 5:**
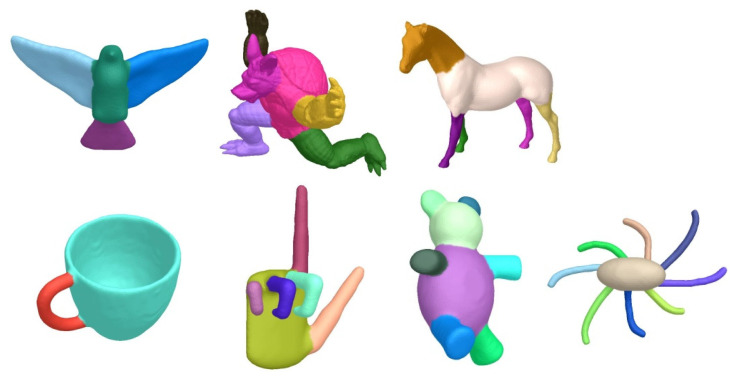
Segmentation results on different kinds of 3D meshes.

Figure 5 demonstrates some segmentation results from the different kinds of 3D meshes, such as a bird, armadillo, horse, cup, hand, teddy, and octopus. Each mesh has a complex shape and specific semantics. It is difficult to work well on all of them. Our algorithm utilizes various shape features effectively and fuses neighboring regions with similar attributes to yield satisfactory results.

### 4.2. Robustness Evaluations

**Figure 6:**
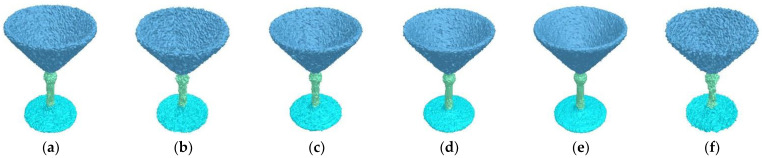
Segmentation results on noisy goblet meshes. The meshes in (**a**–**c**) are corrupted by Gaussian noises along normal directions with intensities of 0.3, 0.4, and 0.5, respectively. The meshes in (**d**–**f**) are corrupted by Gaussian noises along random directions with intensities of 0.3, 0.4, and 0.5, respectively.

**Figure 7:**
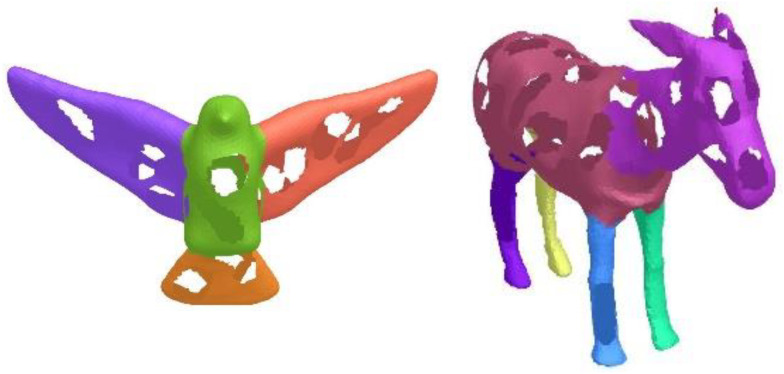
Segmentation results of mesh models with holes.

In this paper, we define the reasonable intra-region difference, inter-region difference, and fusion condition, which are effective and robust. The robustness can be validated by testing meshes with Gaussian noises, holes, missing parts, pose changes, and sampling changes. In Figure 6, we added Gaussian noises to a goblet mesh along normal directions and random directions, respectively. The intensities are 0.3, 0.4, and 0.5. Noise affects the local shape features, and makes mesh segmentation more challenging. Our region fusion method is flexible in dealing with different noises. Therefore, the six results are ideal and consistent with each other. Figure 7 shows the segmentation results for bird and donkey meshes with holes. Each model has many holes of different sizes. In other words, each model has a lot of missing data. The proposed algorithm makes full use of the shape features and can obtain perception-aware results.

**Figure 8:**
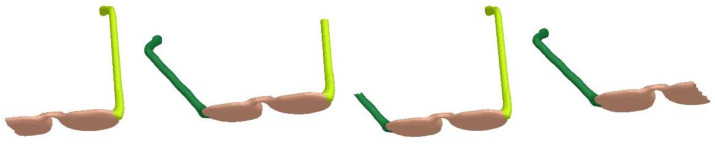
Segmentation results of glasses meshes with missing parts.

**Figure 9:**
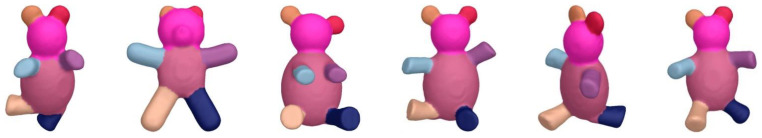
Segmentation results of teddy meshes in different poses.

**Figure 10:**
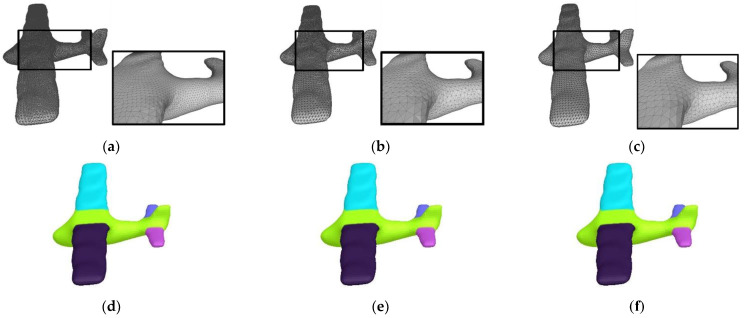
Segmentation results of airplane meshes with different samplings. (**a**) A low-sampling mesh rendered in wireframe (5400 vertices and 10,796 facets). (**b**) A middle-sampling mesh rendered in wireframe (9417 vertices and 18,830 facets). (**c**) A high-sampling mesh rendered in wireframe (19,533 vertices and 39,062 facets). (**d**) Result of (**a**). (**e**) Result of (**b**). (**f**) Result of (**c**).

Figure 8 shows the segmentation results of glasses meshes with missing parts. Although these models are missing different parts, our algorithm can still generate results by complying with semantics. Figure 9 demonstrates the segmentation results of teddy meshes in different poses. This algorithm is oblivious to pose changes, and thus, locates stable segmentation boundaries. In Figure 10, we exhibit the results of airplane meshes with different samplings. Our algorithm is not affected by sampling changes. No matter if the sampling is high or low, these segmentation results are consistent with each other.

### 4.3. Qualitative and Quantitative Comparisons

**Figure 11:**
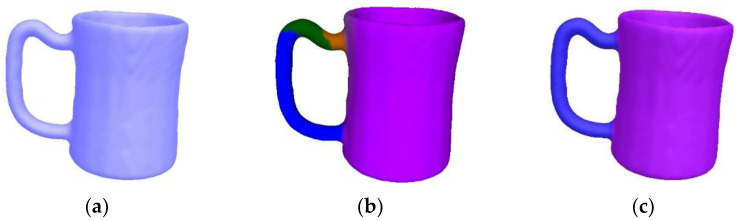
Comparison with automatic segmentation algorithm [2] carried out on a cup mesh. Please note that [2] leads to redundant cuts. (**a**) The original mesh. (**b**) Result of [2] (**c**) Our result.

**Figure 12:**
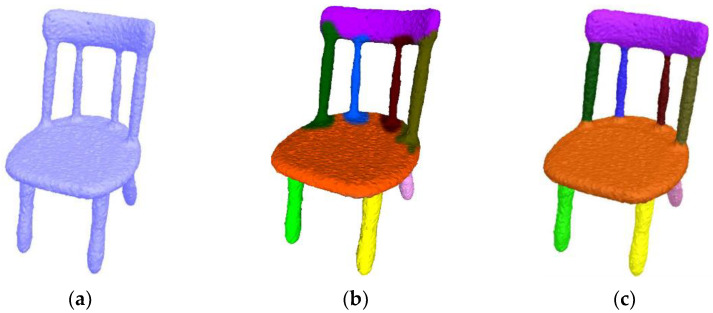
Comparison with deep learning segmentation algorithm [10] carried out on a chair mesh with Gaussian noises along random directions. Please note that [10] is sensitive to noise. (**a**) The original mesh. (**b**) Result of [10]. (**c**) Our result.

Figure 11 compares our algorithm with the automatic segmentation algorithm [2], which depends on the Gaussian curvature and shape concavity. When a mesh model has too many concave creases, redundant cuts occur [2]. Our algorithm fuses local regions of the same part to obtain a better result. Figure 12 shows a comparison between our algorithm and the deep learning segmentation algorithm [10], which utilizes convolutional neural networks and a conditional random field. However, [10] is not robust to noise, thus producing wrong segmentation boundaries. In contrast, our algorithm obtains accurate boundaries.

Quantitative comparisons are also very important. The most intuitive metric is segmentation accuracy, which is defined as the percentage of correctly labeled facets according to ground truth data. Table 1 demonstrates the accuracy comparisons with data-driven segmentation algorithms of [6,9,10,12]. In particular, the algorithms of [9,10,12] are based on deep learning. Data-driven algorithms, especially deep learning ones, are dependent on having a large amount of labeled training data. In practice, their generalization ability and robustness are their bottlenecks. In contrast, our algorithm (i.e., adaptive space partition and iterative region fusion) does not need any training data and is flexible and robust when handling various 3D meshes and complex cases. Therefore, our accuracy is apparently higher than those of [6,9,10,12].

**Figure 13:**
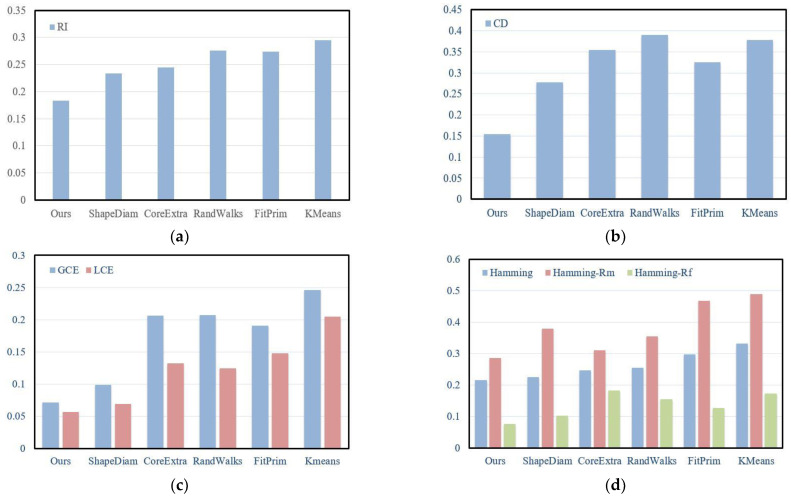
Quantitative comparisons with five state-of-the-art segmentation algorithms of [14,48,49,50,51]. RI is Rand Index, CD is Cut Discrepancy, GCE is Global Consistency Error, LCE is Local Consistency Error, Hamming is Hamming Distance, Hamming-Rm is missing rate, and Hamming-Rf is false alarm rate. Please note that lower values indicate better results. (**a**) Rand Index. (**b**) Cut Discrepancy. (**c**) Consistency Error. (**d**) Hamming Distance.

Chen et al. [22] introduced several metrics for the evaluation of mesh segmentation methods: Rand Index, Cut Discrepancy, Consistency Error, and Hamming Distance. In Figure 13, we further compare our algorithm with five state-of-the-art algorithms quantitatively: ShapeDiam [14], CoreExtra [48], RandWalks [49], FitPrim [50], and KMeans [51]. Our results obtained lower values under these metrics. In other words, our algorithm is superior to these state-of-the-art algorithms.

### 4.4. Further Discussions and Time Performance

**Figure 14:**
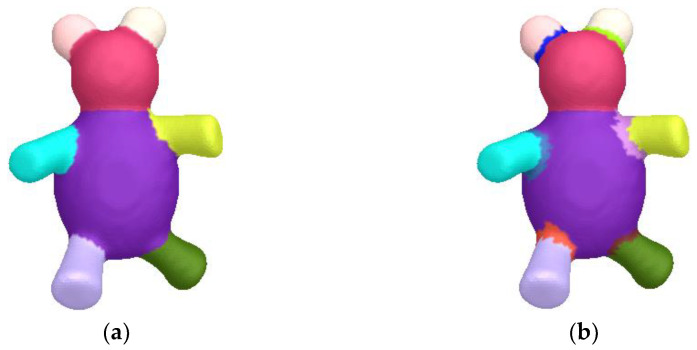
Segmentation results of a teddy mesh with different parameters. Apparent errors appear in (**b**). Please note that the parameters should be set properly to produce a satisfactory result. (**a**) Our result with {$q_{max}$ = 40, $\sigma_{max}$ = 0.1, m = 8}. (**b**) Our result with {$q_{max}$ = 5, $\sigma_{max}$ = 0.05, m = 1.9}.

As with most segmentation methods, the parameters should be adjusted manually for different meshes. In this paper, $q_{max}$ and $\sigma_{max}$ affect over-segmentation, and m affects the fusion condition. We fine-tune these parameters to obtain the best result. Figure 14 demonstrates the segmentation results using different parameters. When m is too small, some regions fail to merge together, thus resulting in apparent errors.

**Figure 15:**
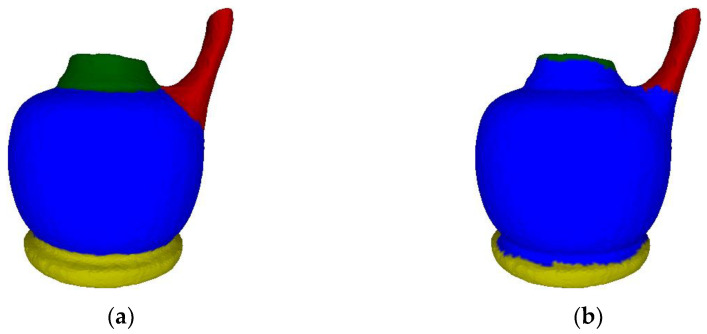
Comparison with our prior paper [43] carried out on an incomplete vase mesh. Please note that [43] generates wrong segmentation boundaries. (**a**) Our result. (**b**) Result of [43].

Our algorithm has substantial improvements over the prior one [43]. We define a new intra-region difference, inter-region difference, and threshold function, which are more reasonable and more flexible for handling different meshes and complex cases. In our prior paper [43], we may have overestimated the difference within a region, and underestimated the difference between adjacent regions. Figure 15 shows a comparison using an incomplete vase mesh that is hard to segment. Some regions belonging to the top part, the handle part, and the base part are fused into the cup part in the result of [43]. Our result looks natural.

Table 2 lists time statistics for some mesh models. The performance depends on several factors, such as the number of facets, the number of superfacets, etc. Overall, our algorithm is very efficient due to binary space partition and region fusion.

## 5. Conclusions and Future Work

This paper introduces a new feature-aware mesh segmentation algorithm. First, a set of superfacets are generated efficiently by using binary space partition. Then, on the basis of superfacets, regions belonging to the same part are fused to obtain the segmentation result. The proposed algorithm makes full use of various shape features and is robust when dealing with different kinds of 3D meshes. Our algorithm can still produce perception-aware segmentation results, even if there are Gaussian noises, holes, missing parts, pose changes, or sampling changes in the mesh models.

The proposed algorithm has several parameters that affect the segmentation results directly. For different mesh models, these parameters are adjusted manually to obtain satisfactory results. We can find suitable parameters automatically through machine learning methods. In addition, the extension of this algorithm to point clouds also deserves further exploration.

## Figures and Tables

**Table 1:** Accuracy comparisons with data-driven segmentation algorithms [6,9,10,12]. Please note that, higher values indicate better results.

Ours	Shapeboost [6]	TOG15 [9]	ShapePFCN [10]	1DCNN [12]
0.9587	0.9371	0.9024	0.9398	0.9362

**Table 2:** Time performance measured in seconds.

Mesh	Number of Vertices	Number of Facets	Number of Superfacets	Time
Airplane	5400	10,796	389	0.0844
Armadillo	25,273	50,542	1311	1.1754
Bird	7849	15,694	587	0.1507
Cup	15,198	30,396	1307	0.2736
Hand	7112	14,220	536	0.1298
Horse	48,485	96,966	1218	1.5426
Octopus	5944	11,888	412	0.1013
Teddy	11,090	22,176	329	0.0964

## Data Availability

Not applicable.
